# More Expert Opinion

**Published:** 1920-04-03

**Authors:** 


### SCRAPPING THE TRAINED NURSE.

### IV.—More Expert Opinion.

Following my interview with Dr. George Peacocke, I sought a further interview with a hospital matron in whose sound judgment I have implicit confidence. I regret that the reader will have to accept my valuation of this lady's worth, as she declines to allow her identity to be revealed. The hospital of which she has charge possesses a deservedly high reputation both as a training-school and as a refuge for the sick poor. I shall refer to this lady as Matron X.

### A Business Proposition.

Matron X is cosmopolitan in her experience, highly accomplished, and thoroughly practical. What I sought particularly to ascertain from her was this : If the Minister of Health were to sanction the equipment of hospitals of a certain standard for the necessary additional training of Health Visitors, and to make them eligible to claim the grants that are now being earned by Polytechnic and other institutions, would hospitals find it in their interests to enter the field ?

I put the question : "Would you, Matron X, in such circumstances, recommend the adoption of the scheme in your hospital? Would you submit it to your Committee as a business proposition?" The answer was an emphatic affirmative. It would benefit the hospital to receive the grants, and the wider outlook for nurses would have the immediate effect of raising the standard of recruiting for the profession. These are the business arguments put forward by Matron X, who states that it is common knowledge that the average standard of candidate probationers all over the kingdom has declined. Weighty arguments!

### The Procedure.

Next, I put it to the Matron : Suppose the Minister of Health made it possible, and that the hospital agreed, how would you carry out the scheme? I admit that the question is scarcely a fair one to spring suddenly on even an experienced matron. Many considerations present themselves. First, there are the relations that exist between hospital and probationer. She must receive her diploma at the end of three years. Matron X gave it as her opinion that if a probationer desired to obtain a Health Visitor's diploma, there ought to be no difficulty in arranging for her to spend a further period of six months in the hospital, specialising and studying and doing a certain amount of nursing at the same time. A nurse could in her third year, she added, make her mind up as to whether or not she would take the Health course. Doubtless the condition of the market at the time would largely determine her choice.

"Now, touching the curriculum," I inquired, "what is there in this over and above the professional education imparted in the ordinary course of a probationer's training in a good hospital?" "Practically nothing," was the reply, "except Maternity, Infant and Child Welfare, and Social Science, all of which might easily be taught to an intelligent Nurse within the space of six months. There are many ways. For example, several hospitals might combine in employing an approved lecturer for Health Visitor candidates. Or, a Women's College or technical institution might provide the necessary instruction at such time and place as would suit nurses."

The hospitals, Matron X insisted, ought to have a chance. It is within their power to provide the best possible candidates. It would pay them in every way to undertake the training. And the Minister of Health, on his part, could always safeguard his Department's interests by awarding grants only on account of candidates who had passed an examination the terms of which he could prescribe. It might include practical, oral, and written tests.

### An Unfair Monopoly.

I am unable to comprehend why, in the first place, a monopoly should be conferred by the State on institutions connected with universities, and, secondly, why a large army of extremely capable and most deserving women should be deliberately prevented from entering the service of the Ministry of Health. Matron X holds that very few Trained Nurses will surrender their employment and start afresh as students. They cannot afford to do so, and where this is not the case, few would have the heart to undertake a new enterprise.

The issue is clear. The Nurse is going to win, or else to be scrapped. The Minister of Health provided her with a Registration Act, and, apparently, the erection of a barrier to prevent her from entering a large field of employment is to be an accompaniment of the gift. What is the attitude on this subject, may I inquire, of the matrons and others who are seeking election on the Executive of the College of Nursing?

IERNE.

